# The francophone network on neglected tropical diseases

**DOI:** 10.1371/journal.pntd.0005738

**Published:** 2017-08-31

**Authors:** Jean Jannin, Philippe Solano, Isadora Quick, Patrice Debre

**Affiliations:** 1 Intensified and Innovative Disease Management, NTD Department, World Health Organisation, Geneva, Switzerland; 2 Institut de Recherche pour le Développement, IRD-Cirad UMR 177 Intertryp, Montpellier, France; 3 Institut thématique Multi Organismes (ITMO) I3M / Institut national de la santé et de la Recherche médicale (Inserm), Paris, France; University of California San Diego School of Medicine, UNITED STATES

## Context

Neglected tropical diseases (NTDs) are communicable diseases that affect the world’s poorest populations living in endemic areas and having no or little access to prevention means, diagnosis, or treatment. WHO recognizes 18 NTDs endemic in 149 countries, affecting approximately a billion people (WHO, http://www.who.int/mediacentre/news/releases/2017/ntd-report/en/). Despite their massive impact, which constitutes the “chronic pandemic of the 21st century” [1], only 0.6% of the global healthcare funding is allocated for controlling these diseases. This imbalance has led to the creation in 2005 of the concept of NTDs for rescuing these diseases from oblivion and to stimulate control efforts besides the “big three” funded by the Global Fund (HIV/AIDS, malaria, tuberculosis) through the objectives of the Millenium Development Goals.

In order to reduce the global impact of NTDs as quickly as possible, WHO defined a roadmap in 2012 (http://www.who.int/neglected_diseases/NTD_RoadMap_2012_Fullversion.pdf) with 2015 and 2020 targets for each disease, which were endorsed by all countries and partners through a specific resolution of the World Health Assembly (WHA Resolution No. 66.12 of 27 May 2013). Additional support and resources to eliminate 10 of the most common NTDs were pledged by “The London Declaration on NTDs” under the initiative of the Bill and Melinda Gates Foundation through massive donations of drugs by pharmaceutical companies and donations by governments and foundations. A specific working group was set up at the G7 level under the German presidency. At the European level, the European and Developing Countries Clinical Trial Partnership (EDCTP) program, initially focusing exclusively on HIV/AIDS, malaria, and tuberculosis (EDCTP1, 2003–2015), now targets NTDs in the framework of its second program, EDCTP2 (2014–2023). NTDs are also now specifically mentioned in the 2030 Health Sustainable Development Goals (SDG No. 3).

## Involvement of France in NTDs

France has a long history in the fight against NTDs. Laveran, Pasteur, Yersin, Muraz, Richet, and Jamot were pioneers in the research and control of NTDs. More recently, the involvement of IRD (Institut de Recherche pour le Développement, formerly ORSTOM) has been key in designing and implementing the Onchocerciasis Control Program (OCP) funded by the World Bank in West Africa, which has had a decisive impact on onchocerciasis control. Some examples of recent involvement of French scientists in the field of NTDs who had a significant impact, together with their partners from disease endemic countries (DECs), include (among many other examples):

- the demonstration that semestrial mass administration of albendazole alone has a marked impact on *Wuchereria bancrofti* endemicity levels, allowing the launch of lymphatic filariasis elimination programs in areas in Congo where loiasis is coendemic [2]
- design of a dengue vaccine by Sanofi-Pasteur [3]
- screening of more than 80,000 individuals and treatment of 350 patients with human African trypanosomiasis (HAT) by the West African Programmes Nationaux de Lutte contre la THA (PNLTHA) and IRD in the framework of the WHO Collaborating Center based in Bobo-Dioulasso between 2009 and 2015. This decrease in HAT incidence in West Africa has made it possible to set the elimination of HAT as a public health problem by 2020 [4, 5], as described in the WHO Roadmap.
- development of anti-tsetse “tiny targets” in collaboration with African scientists and with the Liverpool School of Tropical Medicine, which will contribute to HAT elimination [6, 7]
- description of Buruli ulcer transmission from the environment to humans, and seasonal pattern of the disease in Central Africa [8]
- description of Central Africa’s invasion by the tiger mosquito *Aedes albopictus* and its consequences on virus emergence and the risk of arboviroses expansion [9]

## Launching and organization of the network

The Réseau Francophone sur les Maladies Tropicales Négligées (RFMTN) was officially launched on 8 April 2016 in Montpellier. This network, established under the auspices of the alliance for health and life sciences (AVIESAN), promotes collaboration between French research institutions and gathers research institutions, researchers, medical doctors, NGOs (having operational projects in the field of NTDs), the pharmaceutical industry, foundations, and DEC stakeholders. Its goal is to fill gaps on NTDs thanks to a reinforced contribution of France and to focus on elimination of NTDs. The network seeks to federate French and francophone institutions and individuals working on NTDs and to strengthen relations between NTDs stakeholders. It promotes interinstitutional collaborations on translational research, training, and implementation of elimination projects and aims at raising awareness of NTDs. Finally, it also envisions bridging with other existing European and African NTD networks.

The network, based on individual membership, is also open to associations and scientific societies. It is run by a secretariat hosted by Aviesan and is supported by a scientific and strategic committee comprising the member institutions, DEC stakeholders, industry, and NGOs.

The RFMTN has decided to focus on the “elimination of NTDs,” addressing some key questions:

- How do we define ad hoc and implement control activities in order to sustain the targets of the Roadmap?
- In the context of very low prevalence prevailing when approaching or reaching elimination targets, what can be done to offer a new spectrum of research to scientists in order to develop adequate tools (diagnostics, treatments, vaccines, vector-control tools) adapted to this context? How can industries and national governments be convinced to stay on board and maintain their efforts? How can we avoid the “punishment of success” by convincing donors to continue providing funding when prevalence of diseases is becoming very low?

## First activities

### Mapping exercise

The first achievement of the network as a request from the G7 working group on NTDs was to get a better knowledge of “who is doing what” in France on NTDs. The next step will be to expand to francophone countries. A questionnaire was sent to more than 200 members of the network. The results of this analysis were presented at the G7 working group in Brussels in February 2017.

One of the first observations made after this preliminary analysis confirms the fragmentation of the French scientific and medical community working on NTDs. There are 2 causes of fragmentation: (1) an intrinsic one, as addressing 18 different diseases, which are in addition “neglected,” leads to more than 18 different realities and contexts, and (2) a structural one, reflecting the lack of a dedicated school of tropical medicine in the French NTDs organization. Research and training for NTDs as well as other topics are conducted both in research institutes and universities and in some private institutions and foundations. As a result, the landscape of NTDs activities in France is fragmented, with no apparent coordination or coalition of the research on NTDs except in some dedicated institutions that have structured their research teams around this topic, e.g., in IRD, Pasteur Institute, Cirad, and a few universities. Among the NTDs, researchers working on trypanosomatids, *Aedes*-borne NTDs, and helminths are the most represented.

An additional particularity of the French system of research can be highlighted: most of the researchers are government employees, including expats in DEC working on NTDs, which make the in-kind contribution of the French government significant although hardly visible. Research institutions, universities, and French partnerships (including foundations and NGOs) all have their own dedicated international partnership instruments and platforms that they manage and fund with their partners from DECs. Some of the most known include the Pasteur International Network, the IRD Laboratoire Mixtes Internationaux (LMI), Cirad dispositifs en partenariat (DP), laboratoires internationaux associés (LIA), etc.

### First thematic activity: Universal access to diagnosis of NTDs

As diagnosis is a fundamental step in the access to treatment for patients, the RFMTN decided to address this particular topic, which is far from being efficient and which hampers the efficacy of control programs. Diagnostic issues will also be crucial both when elimination approaches and for post-elimination monitoring. A 2-day think tank meeting on universal access to diagnostics for NTDs took place in Annecy, hosted by the Fondation Mérieux, in March 2017. The objective was to see whether the concept developed around the access to drugs for NTDs could be applied to diagnostics. A list of prerequisites was established including 4 key areas: (1) research, development, and innovation, including major gaps and priorities for research and development; (2) organization and implementation of an efficient system for distribution of diagnostics, including forecast, quality control, shipments, storage, and regulations; (3) training for research and development for local laboratories, central and peripheral health systems, and establishment of ad hoc guidelines; (4) advocacy and fund-raising. Specific work groups will be established to address these 4 areas.

### What’s next?

Taking into account the successes obtained in controlling these diseases presented at the second WHO Partners Meeting in Geneva (http://www.who.int/mediacentre/news/releases/2017/ntd-report/en/), a question that keeps popping up is, do we still need science for these diseases?

As responses to diseases move towards the endgame, evaluation and monitoring to ensure post-control surveillance will become critical. The fourth WHO report on NTDs concludes that besides ensuring delivery of health services, eliminating transmission of NTDs constitutes the major challenge beyond 2020 and that “continued efforts are required to ensure treatments are implemented efficiently and that monitoring and surveillance tools are improved, to seek alternative medicines in the event of loss of efficacy or development of resistances, to ensure that reporting systems are effective, and to maintain optimal levels of coverage” [10]. Future efforts in vector control and One Health approach will be decisive to reach the Roadmap objectives [11, 12]. The more we approach elimination, the more interventions have to focus where they are really needed, using context-specific elimination tools—there will not be 1 elimination per disease, there will be as many eliminations as there are local contexts.

One of the future challenges will also be to integrate NTDs into global health as a step towards universal health coverage (UHC) and in this debate, a francophone vision may prove useful [13]. UHC is at the heart of the SDGs and is a cross-cutting issue for many SDGs [10, 14]. The RFMTN considers that elimination of NTDs participates in ensuring fundamental human rights, and elimination of these NTDs is a mandatory step towards addressing SDGs, thus progressing towards UHC. The RFMTN will continue its internal analysis in order to build its vision, taking into account what already exists, welcoming new French and francophone members, and working with other existing NTD networks to promote collaborative partnerships.
